# Change in the faunal composition of mosquitoes (Diptera: Culicidae) along a heterogeneous landscape gradient in the Brazilian Amazon

**DOI:** 10.1371/journal.pone.0288646

**Published:** 2023-07-13

**Authors:** Jessica Feijó Almeida, Heliana Christy Matos Belchior, Fernando Antonio Jutahy Colares Batista, Rebeca Cristina de Souza Guimarães, Ahana Maitra, Claudia María Ríos Velásquez, Thiago Junqueira Izzo, Felipe Arley Costa Pessoa

**Affiliations:** 1 Laboratório de Ecologia e Doenças Transmissíveis na Amazônia, Instituto Leônidas e Maria Deane Fiocruz Amazônia, Manaus, Amazonas, Brazil; 2 Programa de Pós-Graduação em Entomologia, Instituto Nacional de Pesquisas da Amazônia, Manaus, Amazonas, Brazil; 3 Programa de Pós-Graduação em Biologia da Interação Patógeno-Hospedeiro, Instituto Leônidas e Maria Deane Fiocruz Amazônia, Manaus, Amazonas, Brazil; 4 Universidade Federal de Mato Grosso, Cuiabá, Mato Grosso, Brazil; Universidade Federal de Mato Grosso do Sul, BRAZIL

## Abstract

This study aimed to evaluate the influence of different anthropic landscape profiles on the diversity and distribution of mosquito species in a rural settlement of the Brazilian Amazon. Eight field collections were conducted at 18 sampling points interspersed throughout 2020–2021. Plastic containers, bamboo internodes, and tires were used as traps to capture immature mosquitoes in three distinct habitats: forest, forest edge, and peridomicile. A total of 15,547 individuals, distributed in 26 species of culicids, were collected. The most abundant species were *Culex urichii* (8,376 specimens), *Culex* (*Melanoconion*) (2,473 specimens), and *Aedes albopictus* (1,252 specimens). Forest habitat showed the highest abundance, and forest edge showed the highest species richness. Different types of environments influenced both the abundance and richness of mosquitoes. The species composition was also significantly different between the analyzed sites, mainly between forest and peridomicile environments. The change in species dominance could largely explain this change in mosquito community composition. *Haemagogus janthinomys*, an important sylvatic arbovirus vector, was found in peridomicile habitats and *Ae*. *albopictus*, a vector associated with human environments, was found in forest habitats, thus providing evidence of species spillover. Our results indicated that landscape changes affect mosquito communities, influencing their richness and abundance. These changes may have implications for future arboviral outbreaks in this rural settlement due to the possible establishment of sylvatic vector species in anthropic environments.

## Introduction

Mosquitoes belonging to the family Culicidae are widely distributed across the globe. Much of this success is related to its ability to colonize the most different types of habitats. In their immature stage, they colonize natural and/or artificial aquatic environments of different types and sizes. For example, the Amazonian sylvatic mosquitoes of the Anophelini tribe are usually found in lakes, rivers, and fish ponds, while those of the Sabethini tribe develop in phytotelms [1, 2]. Other Amazonian species, such as those belonging to the Culicini and Aedini tribes, are eclectic regarding breeding grounds and can be found in ponds, artificial containers that accumulate water, holes in trees, and others [3]. Regarding their importance in the aquatic food chain, mosquito larvae are food for dragonflies, fish, and beetle species [4].

Mosquitoes are notably one of the main focuses of studies about emerging and re-emerging diseases worldwide. A significant fraction of the 3,614 existing mosquito species [5] are vectors of various arboviruses and other parasites that cause millions of infections and deaths in humans, mainly in tropical and subtropical regions. For example, in Brazil, severe outbreaks of arboviruses, such as Dengue (1,172,882 probable cases and 585 deaths), Zika (5,699 probable cases), Chikungunya (122,075 probable cases and 23 deaths), Yellow Fever (1,267 suspected epizootics and four deaths), have been recorded in recent years; however, the impact of co-circulating arboviruses is still unknown [6, 7]. Multifactorial causes are responsible for these outbreaks; among such factors is the change in the composition of the vector fauna caused by anthropic actions.

Several vectors of human diseases, particularly the Culicidae, are sensitive to anthropic environmental changes. When the environment is impacted, the mosquitoes suffer changes in their abundance and diversity, and in many cases, the type of landscape is directly related to their distribution and the occurrence of their hosts [8]. In addition, anthropogenic activities often result in modified habitats exhibiting various environment filter differences. Deforested habitats, for instance, are characterized by higher temperatures and greater light intensity, which can limit the occurrence of more sensitive species [9]. Nevertheless, these same conditions can reduce the larval development time for certain mosquito species [10]. Furthermore, due to the environmental alterations caused by anthropogenic activities, there is often irregular and intense rainfall, which, combined with the increased availability of human-made structures that collect water (such as fish farming tanks, water storage tanks, and improperly disposed of trash), can provide a more diverse range of oviposition sites.

Habitat modification can generate different scenarios of mosquito Beta-diversity within a landscape. Several non-random local extinctions of habitat-specialist, or more sensible species, can show us a pattern where the community of the "new" habitats is a subset of the original habitat (nested pattern) [11]. The community of the modified habitat can receive new species by migrating from other habitats, generating a nested pattern [12]. Both mechanisms of pattern generation through nestedness do not necessarily exclude one another, and immigrant species can potentially replace locally extinct species or even cause their extinction, leading to a community with high species turnover [11, 13]. Chaves et al. [14] verified the association between the percentage of vegetation cover and the diversity of Amazonian mosquitoes and found that, in general, the decrease in vegetation cover led to the loss of Culicidae diversity, however, *Anopheles darlingi*, the primary vector of malaria in the region, has become dominant in the most anthropized areas.

Both nested and turnover patterns of Beta diversity are not excluded within a landscape. Some studies have identified the ecotone between forested and anthropic areas to host more species of Culicidae because they harbor both: species typical of forests and habitat-generalist commonly found in anthropized areas [15, 16]. So, anthropization can generate a complex landscape, increasing the Beta-diversity among different landscape unities. This higher diversity can influence the transmission of mosquito-borne diseases since sylvatic mosquito vectors are frequently found in these places [17]. Vieira et al. [18] identified sylvatic vectors *Cx*. *declarator* and *Cx*. *(Melanoconion)* spp. in urbanized areas of a region in the Southern Amazon. In addition to this evidence of apparent modification of the fauna in response to anthropization, there is also concern regarding the spillover phenomenon, where pathogens such as arboviruses, which were previously restricted to the sylvatic cycle, start circulating in previously uninfected human population, through bridge vectors [19–21]. Identifying such a phenomenon is a crucial factor in predicting future outbreaks.

On a more local scale, people living in rural Amazonian settlements are constantly affected by mosquito-borne diseases, which might be an effect of landscape modification due to sustenance practices, such as deforestation for subsistence agriculture or extensive livestock farming, fish farming ponds, hunting, and others [22, 23]. As the type of landscape can influence the mosquito community, our study primarily aimed to compare the abundance, richness, and patterns of the composition of mosquitoes among the forest, forest edge, and peridomicile habitat in small farms of Brazilian Central Amazon, and also identify the species composition of each habitat.

We hypothesize that different mosquito species will react differently to their habitat changes. More generalist mosquito species, which can use various breeding sites, possess a varied diet, and withstand sudden abiotic changes, will likely colonize diverse environments. Conversely, species with more restricted breeding habits will show a restricted distribution within our sample sites, limited to specific environments, such as more forested or anthropized areas. Based on this, we predict that: 1) the richness and the abundance of species will be higher on the edge of the forest; 2) the three habitats will show a different mosquito composition. When considering the causes for the potential differences in mosquito species composition, we infer that: 2.1) most of the variation will be due to the replacement of species between habitats (turnover), and; 2.2) this will result in different indicator species to each habitat type. The variation in mosquito species composition between environments due to nesting will only account for a small portion of the mosquito population.

## Methods

### Study area

**Fig 1:**
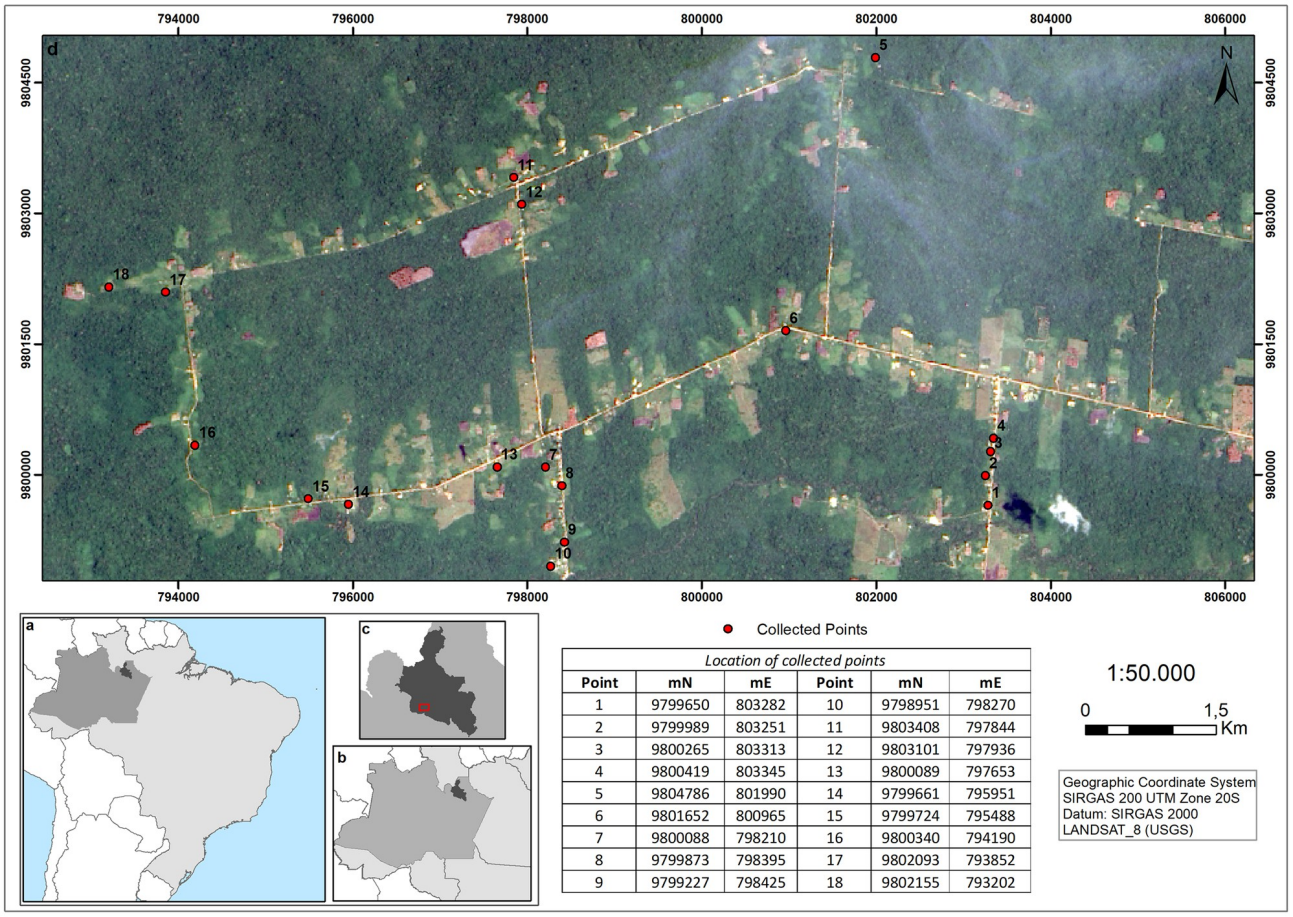
Location of the study area. a–Brazil. b–State of Amazonas. c—Municipality of Presidente Figueiredo and highlighted in red, rural settlement Rio Pardo. d–distribution of collection points (in red) along the rural settlement of Rio Pardo. The numbers represent the geographic coordinates of the collection site. mN–meters north. mE–meters east.

Mosquitoes for this study were collected in a rural settlement of Rio Pardo (S01º49’02.4" W060º19’03.6"), municipality of Presidente Figueiredo, in the state of Amazonas, Brazil. The settlement is located in a dense ombrophilous terra firme forest bordering the Canoas Settlement, Waimiri–Atroari Indigenous Reserve, and private or Brazilian government lands. Rio Pardo was officially created in 1996 through the National Institute for Colonization of Agrarian Reform and comprised an area of about 28,000 hectares, approximately 550 inhabitants, and around 170 households. The settlement consists of 6 unpaved roads, wooden houses, monoculture plantations, and fish ponds. According to the Köppen-Geiger classification, the climate in the area is moist tropical [24], with an average annual temperature of 27°C and two climatic seasons: rainy (November to May) and dry (June to October) (Fig 1).

From a satellite image of IKONOS^™^, 18 sampling points were randomly selected. Each point was delimited by a 200-meter buffer and covered three different environments: forest (area with vast preserved and non-flooded vegetation—"terra firme" rain forest); forest edge (intermediate region, between the anthropized site and the natural vegetation fragment); and peridomicile (surrounding the residence, having an orchard, vegetable garden and livestock like chickens, pigs, dogs, and others).

### Mosquito collection

Eight collections were interspersed between 2020 and 2021 (S1 Table). To capture a greater diversity of immatures, we used three types of larvitraps (bamboo internodes, plastic containers, and tires) (S1 Fig). The bamboo internode container was made by cutting the top opening of each internode, thus, facilitating the removal of the collected material. The tires were received as donations from tire stores and cut in halves. After the confection, all larvitraps were sanitized to avoid contamination. In each type of environment, one unit of each kind of larvitraps was installed so that in the end, each sampling point had nine larvitraps (1 x 3 x 3) and a total of 162 larvitraps (18 x 3 x 3) per collection event. The larvitraps were installed 1 m above the ground and filled with about 500 ml of untreated well water. Fifteen days after installation, all water and the organic material were removed from each larvitraps, inserted in individual plastic bags, duly labeled, and the larvitraps were refilled with water. In the laboratory, the collected material was sorted to separate possible predators, and the mosquito larvae were reared in plastic pots containing water and organic material obtained from the larvitraps at the time of collection. The emerged adults were stored in a -20 refrigerator for later identification with dichotomous keys [25, 26]. The nomenclature used in this study was according to [27].

The conducted study was authorized by the Research Ethics Committee of Instituto Nacional de Pesquisas da Amazônia (CEP nº 30818220.6.0000.0006) and Sistema de Autorização e Informação em Biodiversidade—Br (SISBIO-ICMBio nº 75057–1). We declare that all participants were instructed, agreed to participate in the study, and signed a written consent form. This work did not include minors.

### Data analysis

To evaluate the effects of environments on mosquito abundance of individuals and the number of species, we used Generalized Linear Mixed Models (GLMM) with Gaussian distribution (for normally distributed abundance values) and Poison distribution (indicated to count data). The type of trap installed at the sites (1|trap/site) was used as a random factor in the model. The *lme4* and *lmerTest* packages were used to perform the GLMMs and evaluated the adequacy and overdispersion using the *DHARMa* package [28–30]. The rarefaction curve, based on individuals, was constructed to determine whether the number of mosquitoes collected in the environments reached the species richness saturation point. To evaluate the influence of environments on mosquito composition, we applied a Permutational Multivariate Analysis of Variance (PERMANOVA) with the *vegan* package [31]. After detecting significant differences (p<0.05), we performed a paired PERMANOVA (*pairwiseAdonis* package) [32] to identify the environment(s) where this difference was observed. We have also used a Nonmetric Multidimensional Scaling (NMDS) to spatially represent the species diversity profiles of each environment with the *ggplot2* and *vegan* package’s functions, ’adonis’ and ’metaMDS’ [31, 33].

To determine whether the dissimilarity in species composition could be explained by species substitution or nestedness, we applied the additive partitioning of beta diversity using the *betapart* 1.5.6 package [34]. We used the qualitative data matrix to calculate the beta diversity using the Sørensen dissimilarity index (βSOR), partitioned into beta Simpson (βSIM), which indicated species substitution, and beta nestedness (βNES), which quantified subsets of communities. To verify the possible fidelity and specificity of species to specific environments, the species indication value (IndVal) was obtained with the *labdsv* package [35]. Except for the rarefaction curve, which was performed using the Past 4.09 program [36], all other statistical analyses were performed using the R software [37].

## Results

A total of 15,547 individuals were collected, which were distributed among 26 species of immature mosquitoes. Among the environments, the forest had a greater abundance of mosquitoes, with 5,555 individuals, followed by forest edge, with 5,428 individuals, and peridomicile, with 4,564 individuals. Forest edge showed a higher species richness with 26 species, while in forest and peridomicile, the richness of immature mosquitoes was in both, represented by 20 species (Table 1).

**Table 1:** Diversity of mosquitoes. Immature mosquitoes collected in different environments of the rural settlement of Rio Pardo, Presidente Figueiredo, Amazonas, Brazil, during 2020–2021.

Species	Forest	Forest edge	Peridomicile	Individuals total
*Aedes albopictus*	18	33	1201	1252
*Culex (Melanoconion)* sp.	1164	927	382	2473
*Culex nigripalpus*	14	17	30	61
*Culex quinquefasciatus*	0	23	0	23
*Culex urichii*	3662	3097	1617	8376
*Haemagogus janthinomys*	26	70	122	218
*Haemagogus leucocelaenus*	2	22	5	29
*Limatus durhamii*	103	181	300	584
*Limatus flavisetosus*	42	40	100	182
*Limatus pseudomethysticus*	10	27	14	51
*Ochlerotatus argyrothorax*	21	18	1	40
*Orthopodomyia fascipes*	225	477	31	733
*Sabethes albiprivus*	3	6	2	11
*Sabethes bipartipes*	4	13	101	118
*Sabethes cyaneus*	0	4	0	4
*Sabethes glaucodaemon*	2	17	30	49
*Sabethes tarsopus*	0	1	0	1
*Sabethes tridentatus*	0	1	23	24
*Toxorhynchites bambusicolus*	29	21	14	64
*Toxorhynchites haemorrhoidalis*	30	48	161	239
*Trichoprosopon digitatum*	195	351	421	967
*Wyeomyia aponoroma*	2	14	2	18
*Wyeomyia* sp1	2	3	0	5
*Wyeomyia* sp2	0	4	0	4
*Wyeomyia* sp3	1	2	0	3
*Wyeomyia* sp4	0	11	7	18
**Individuals Total**	**5555**	**5428**	**4564**	**15547**

In all environments, the most abundant species was *Culex urichii*, representing 65.92% of the total specimens collected in the forest, 57.06% in the forest edge, and 35.43% in the peridomicile. The second and third most abundant species in forest and forest edge were, respectively, *Cx*. (*Melanoconion*) sp. (20.95% in forest, 17.08% in forest edge) and *Orthopodomyia fascipes* (4.05% in forest, 8.79% in forest edge). In addition to *Cx*. *urichii*, the other most abundant species in the peridomicile, were *Aedes albopictus* (26.31%) and *Trichoprosopon digitatum* (9.22%).

**Fig 2:**
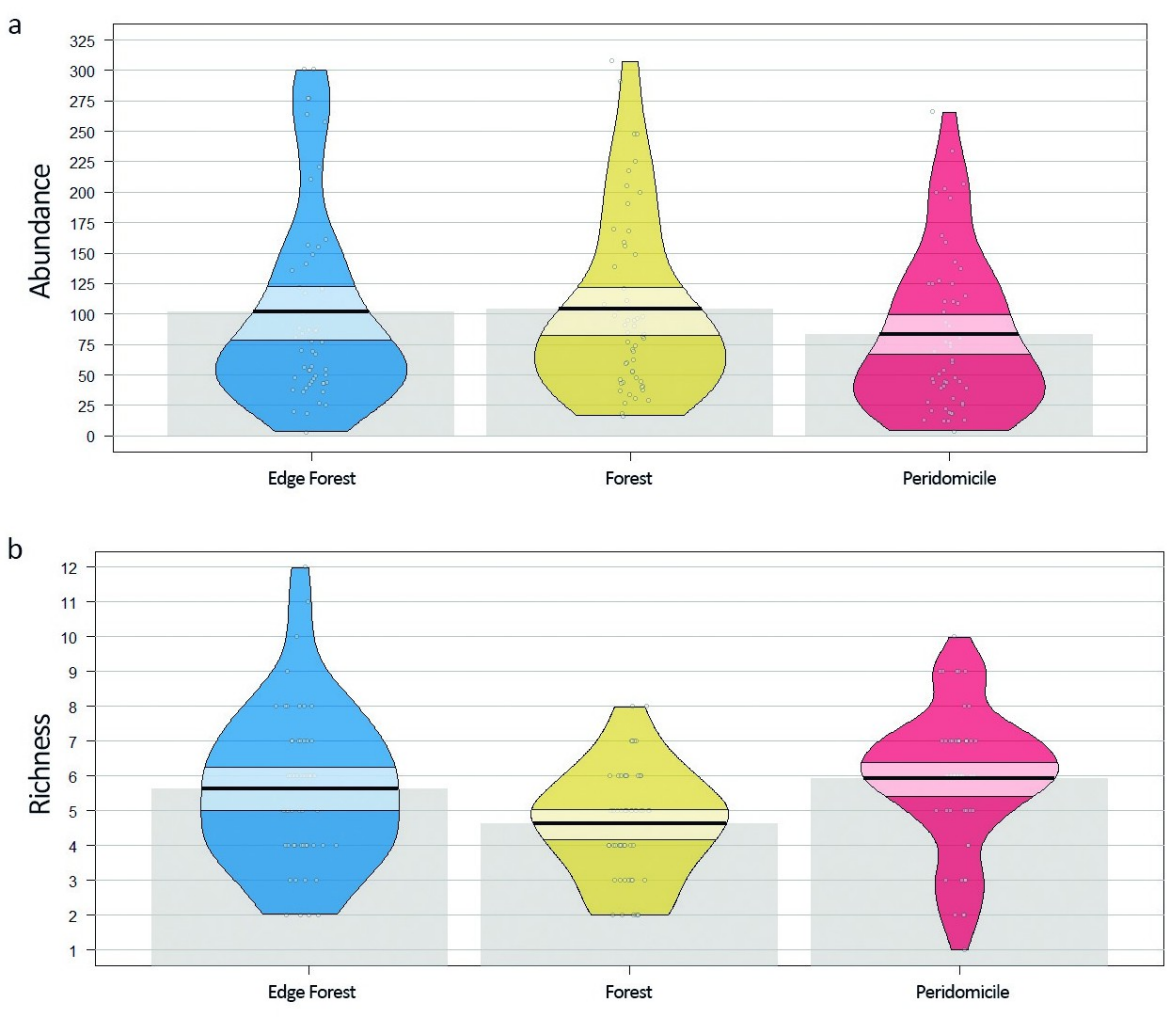
Abundance (a) and richness (b) of mosquitoes identified in three types of environments in the rural settlement of Rio Pardo, Presidente Figueiredo, Amazonas, Brazil. Boxplots represent the maximum, minimum, and mean, with a 95% confidence interval. Points correspond to raw data.

The abundance (x^2^ = 5.6088, df = 2, p = 0.06) and richness (x^2^ = 9.1881, df = 2, p = 0.01) of mosquitoes were influenced by the difference among studied environments. Forest edge and forest showed similar abundance averages (± 110 individuals), while peridomicile averaged ± 80. As for richness, peridomicile and forest edge were closer in their averages (± 5.8 species), whereas forest showed the lowest average (± 4.8 species) (Fig 2).

**Fig 3:**
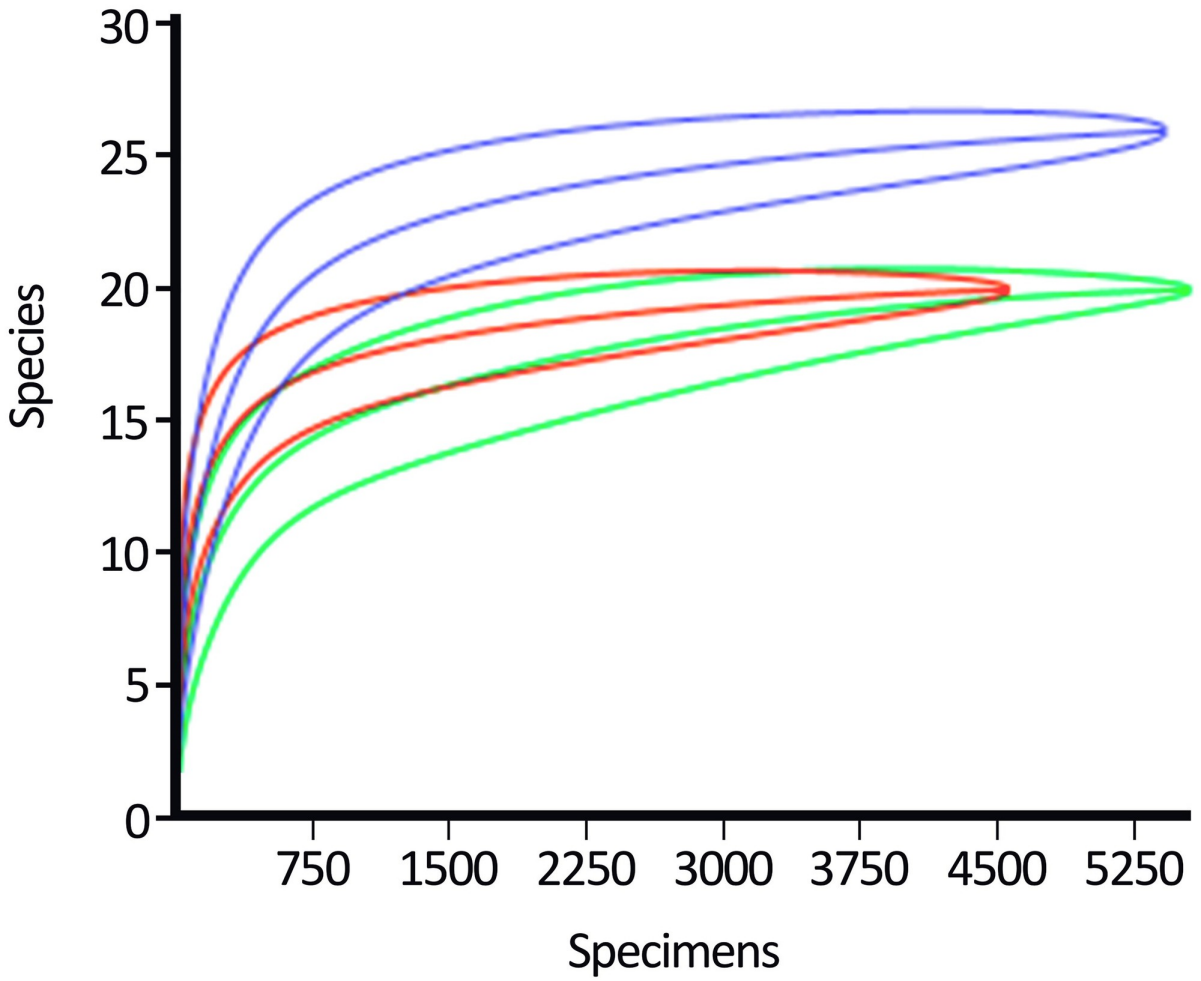
Mosquitoes collection. Rarefaction curve of species collected in the forest (green line), forest edge (purple line), and peridomicile (red line) environments of the Rio Pardo settlement, Presidente Figueiredo, Amazonas, Brazil.

It was possible to obtain the asymptote in the rarefaction curve of the species in all environments. For the forest edge, species diversity started being different from 1,500 specimens upwards compared to the other environments. Forest and peridomicile showed relative approximations in their means and standard deviation of species diversity (Fig 3).

**Fig 4:**
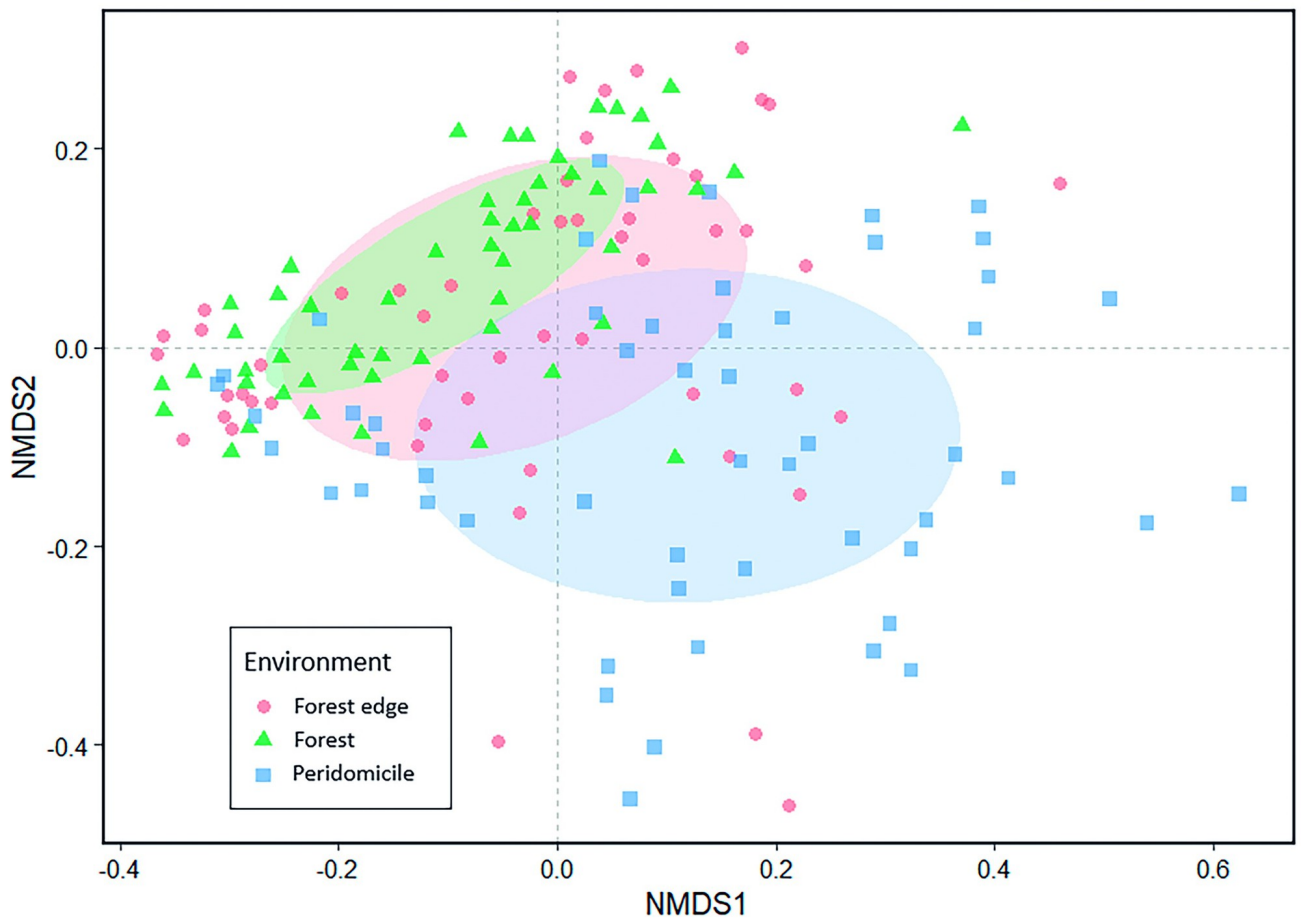
Nonmetric Multidimensional Scaling (NMDS) comparing mosquito species composition between forest, forest edge, and peridomicile environments.

The species occurrence between the three environments differed significantly (PERMANOVA r^2^ = 0.12868, F 11.668, p = 2e-04). The paired PERMANOVA test showed that all environments were statistically distinct from each other: Forest edge x Forest (r^2^ = 0.02684, F 2.8964, p = 0.025); Forest edge x Peridomicile (r^2^ = 0.08008, F 9.1401, p = 0.001); Forest x Peridomicile (r^2^ = 0.17611, F 22.6527, p = 0.001). The spatial distribution of species frequency among the three environments showed more significant dissimilarity between the peridomicile and forest. In contrast, the forest edge was more similar to the forest (Fig 4). In general, when evaluating the composition from the occurrence of species, the additive partition of β_SOR_ (β_SOR_, mean = 0,5400838) showed that 73% of the dissimilarity was due to species substitution among sampling points (turnover, mean = 0,3974169) and just 27% was due to difference in richness among sampling points (nesting, mean = 0,142667).

Eleven species were identified as potential bioindicators. *Orthopodomyia fascipes* and *Wyeomyia aponoroma* showed greater specificity and fidelity to the forest edge environment. *Culex* (*Mel*.) sp. and *Cx*. *urichii* were the bioindicators of forest habitats. *Aedes albopictus*, *Toxorhynchites haemorrhoidalis*, *Sabethes bipartipes*, *Limatus durhamii*, *Haemagogus janthinomys*, *Sa*. *tridentatus* and *Sa*. *glaucodaemon* were associated with the peridomicile environment (Table 2).

**Table 2:** Specificity and fidelity of mosquitoes of the forest, forest edge, and peridomicile collected during 2020–2021 in Rio Pardo, Presidente Figueiredo, Amazonas, Brazil.

Species	Environment	IndVal%	*p-*value	Frequency
*Orthopodomyia fascipes*	Forest edge	26	0.015	39
*Wyeomyia aponoroma*	Forest edge	11	0.014	9
*Culex* (*Melanoconion*) sp.	Forest	44	0.004	129
*Culex urichii*	Forest	44	0.016	123
*Aedes albopictus*	Peridomicile	79	0.001	59
*Toxorhynchites haemorrhoidalis*	Peridomicile	55	0.001	83
*Sabethes bipartipes*	Peridomicile	42	0.001	30
*Limatus durhamii*	Peridomicile	33	0.019	71
*Haemagogus janthinomys*	Peridomicile	26	0.036	58
*Sabethes tridentatus*	Peridomicile	20	0.001	11
*Sabethes glaucodaemon*	Peridomicile	16	0.025	24

## Discussion

Our study in an area of recent settlement in Central Amazonia suggested that habitat anthropization may cause variation in the abundance, richness, and composition of mosquito species. These results have several significant consequences from ecological and public health points of view. We observed that the substitution of mosquito fauna (turnover) is the main component of the beta diversity associated with the landscape changes. So, the effect of anthropic modification on landscape generates "new" habitats, permitting the establishment or favoring the survival of those species which were not common to the forest. This causes a scenario where a given person on the deforestation frontier faces three different mosquito fauna associated with each habitat, separated only by a few meters: the peridomicile, the forest edge, and the forest. Thus, this person is consequently subjected to a greater variety of diseases transmitted by mosquito vectors.

The species collected in this study are relatively common throughout the Amazon region and, for example, represent 45% of the total species richness recorded in Rio Pardo [15]. As these are micro-breeding sites, it was predictable that most of the mosquitoes collected were associated with phytotelma, which reduced the richness of mosquitoes in this study. However, our rarefaction curve proved that it was possible to collect at least the most common species from the analyzed sites based on our capture method. Other studies conducted in the Brazilian Amazon have shown difficulties in sampling until mosquito richness is saturated, especially when dealing with forest environments [38, 39]. The use of multiple collection methods can maximize the capture of different Culicidae species. One of the latest inventories to record a high richness of Amazonian species was carried out by Hutchings et al. [40], where, through capturing adult and immature mosquitoes with different types of traps, the authors successfully identified 112 species of mosquitoes.

Being the most abundant species in the study, *Cx*. *urichii* represented more than half of the collected mosquitoes and was also present in all environments. In our previous study [41], *Cx*. *urichii* was also classified as the most abundant species, but in other surveys of regional fauna, this species was observed in low abundance [42, 43]. This species is generally present in primary and secondary forest environments and, so far, has not been associated with arboviruses [44, 45]. *Culex* (*Mel*.) and *Or*. *fascipes* were observed to be in greater abundance in the forest habitat. It was not possible to identify the mosquitoes of the subgenus *Melanoconion* at the species level, as it is a species complex, but in general, these culicids are present in various environments, ranging from preserved to highly anthropized sites [46, 47]. Some species of *Cx*. (*Mel*.) participate in the natural cycle of arboviruses, including Venezuelan Equine Encephalitis and West Nile virus. However, *Or*. *fascipes* is a primarily sylvatic mosquito with no medical importance [48]. In the peridomicile habitat, the most abundant mosquitoes were found to be *Ae*. *albopictus* and *Tr*. *digitatum*. Both *Ae*. *albopictus* and *Tr*. *digitatum* are associated with anthropic environments and are vectors of several arboviruses [49, 50]. Therefore, the abundance of such species in the peridomicile environment represents a high risk of arbovirus outbreak for people living in this settlement.

This study observed the effect of the environment on mosquito abundance and species richness. The averages and absolute values of abundance and species richness in the environments indicated that the most preserved and transitional locations had higher abundances, whereas the richness was higher in transitional and anthropized areas (Fig 2). Forest edge, classified as a transition environment, was associated with higher abundance and species richness. The change in the mosquito richness and abundance pattern is generally correlated; preserved areas with greater richness and anthropized areas with greater abundance have been reported in some studies (e.g., [16, 51, 52]). In the Southern Brazilian Amazon, Vieira et al. [53] reported differences in mosquito abundance and richness between different sites and observed that abundance and richness were positively correlated with disturbed landscapes. Due to the particularity of each study, such as sampled locations, collection method, and others, it isn’t easy to measure what may be leading to this difference in abundance and richness between preserved and anthropic environments.

It was possible to identify a difference in the composition of the mosquito community, as predicted in our hypothesis. The group of species that composed each environment was somewhat expected, for example, *Ae*. *albopictus* and *Li*. *durhamii* were correlated to the peridomicile; these species are usually known to have preferences for the most anthropic sites [50, 54]. However, mosquitoes, like *Hg*. *janthinomys* and some species of the genus *Sabethes*, usually known as sylvatic, were observed to be associated with the anthropic environment. Other studies have also reported the presence *Hg*. *janthinomys* in forest edges, open fields, and urban/built-up land cover [55, 56]. We believe that the alteration of its natural habitat due to deforestation, associated with food eclecticism [57], and its ability to fly great distances [58] may cause the successful colonization of *Hg*. *janthinomys* in new environments. As for *Sabethes* species, despite being associated with anthropized environments in this study, such results need to be further investigated since the general abundance of these species was low. However, Hendy et al. [59] propose that due to the ability of *Sabethes* species to reproduce in a wide variety of phytotelms, they can establish themselves on the forest edges.

*Orthopodomyia fascipes* and *Wy*. *aponorama*, which are usually frequent in preserved environments with their immatures developing into phytotelmata [25, 60], were associated with the forest edge environment in this work. Although we have not evaluated other factors related to the presence of these species, we suggest that the microbreeding sites found at the forest edge, as well as the greater intensity of light and higher temperature, when compared to the interior of the forest, could be promoting the establishment of *Or*. *fascipes* and *Wy*. *aponorama* in these environments. The difference in the composition of the culicids community in our investigation area was primarily associated with the change of species among the sampling points concerning the environments analyzed (see Results). The turnover in the mosquito community due to landscape gradients is routinely observed in other studies [13, 61–63] and is generally influenced by the characteristics of the local habitat. In this case, associated anthropogenic species are favored and replace the peridomicile’s typical local fauna. Also, the occurrence of some species is favored in the forest edge. The mechanisms of such replacement are still unknown, but we suggest they are associated with factors such as ecological filtering, competition, predation, and others [9, 64]. So, based on our results, we can say that there are three locally adapted non-independent communities, one for each studied habitat. The occurrence and abundance of typically sylvatic vector species in anthropic environments, such as those identified here (Table 1), maybe a worrying factor and possibly lead to an increase in human-vector contact [65, 66]. The presence of anthropic vectors, e.g., *Ae*. *albopictus*, in forest edges and forests, also impose a risk of infection of viruses in each habitat (Table 1).

From an epidemiological point of view, the specificity of sylvatic vectors in the peridomiciliary environment represents an additional aggravating factor for rural inhabitants, who generally live in conditions of social vulnerability in the settlement. As favorable conditions were created for establishing sylvatic species in anthropic environments, especially vectors, local arboviral outbreaks could possibly emerge and re-emerge. The high seroprevalence of the *Mayaro* virus in children and women from Rio Pardo, who usually did not frequent the forest, raised the hypothesis that this arbovirus was possibly being transmitted by other vectors [67]. However, based on our results (see also [41]), we cannot rule out the possibility that the primary vector, *Hg*. *janthinomys* is involved in the transmission cycle of this virus in this settlement.

Our study corroborated our hypotheses and provided evidence that anthropogenic changes influence the Amazonian mosquito community. We showed that species richness and abundance of mosquitoes were correlated with habitat type. The difference in the composition of the mosquito fauna is primarily related to the substitution of the dominant species among different environments (turnover). The presence and higher abundance of notorious vector species, such as *Ae*. *albopictus* and the other indicators species, in the peridomicile environment, associated with favorable conditions for their establishment and survival (e.g., access to food resources and a variety of breeding sites), increases the risk of circulating arboviruses. Such observations reinforce the need for further exploratory field studies regarding the landscape features and their modifications that may shape the mosquito community, as environmental changes arising from anthropic factors significantly impact the diversity of mosquitoes.

## Supporting information

S1 Fig Immature traps used for mosquito collections, in 2020–2021, in the rural settlement Rio Pardo, Presidente Figueiredo, Amazonas, Brazil. Highlighted, a–Bamboo internode, b–Tire, c–Plastic container.
(TIF)

S1 Table Data from mosquito collections carried out in 2020–2021 in the rural settlement Rio Pardo, Presidente Figueiredo, Amazonas, Brazil. (XLSX)
