# Supplementary figures and images for: Change in the faunal composition of mosquitoes (Diptera: Culicidae) along a heterogeneous landscape gradient in the Brazilian Amazon
Source: PLoS One. 2023 Jul 13;18(7):e0288646. doi: 10.1371/journal.pone.0288646 (PMC10343073; doi:10.1371/journal.pone.0288646)

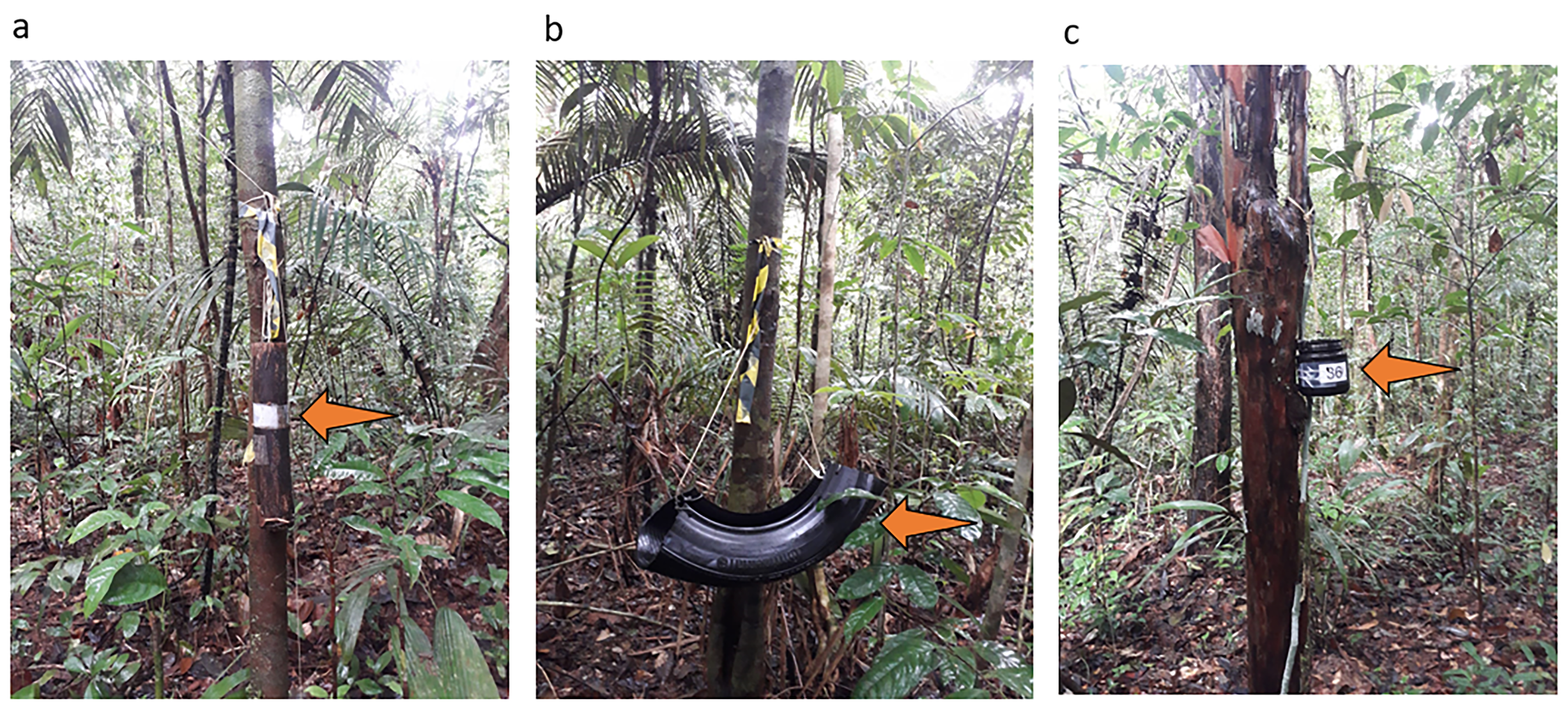

Supplement: S1 Fig — Highlighted, a–Bamboo internode, b–Tire, c–Plastic container. (TIF) [file pone.0288646.s001.tif]
